# A Truncation in the Regulator RocA Underlies Heightened Capsule Expression in Serotype M3 Group A Streptococci

**DOI:** 10.1128/IAI.02892-14

**Published:** 2015-03-17

**Authors:** N. N. Lynskey, C. E. Turner, Li S. Heng, S. Sriskandan

**Affiliations:** Faculty of Medicine, Imperial College London, Hammersmith Hospital, London, United Kingdom

## LETTER

The recent report from Cao et al. regarding hyper-virulence associated with M3 group A streptococcal (GAS) infections identified serotype-specific mutations in two important gene regulators, *fasC* and *rivR*. These mutations were demonstrated to underlie the differential expression of two important virulence factors, streptokinase and protein G-related 2-macroglobulin-binding (GRAB) protein, respectively. However, the observed heightened expression of the hyaluronic acid capsule in M3 GAS could not be attributed to these mutations, despite RivR being a known regulator of this important virulence factor (1).

**FIG 1:**
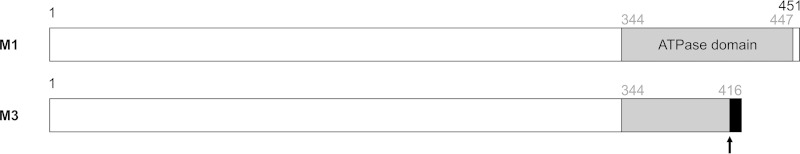
Schematic of serotype M3 RocA truncation. Linear representations of M1 and M3 RocA. The gray bars denote the precise location of the predicted ATPase domain. The black arrow indicates the location of the conserved frameshift mutation at codon 410 of M3 RocA. The black box represents a subsequent sequence alteration and a premature stop codon which results in the truncation of the M3 RocA protein at amino acid 416.

Hyper-encapsulation of serotype M18 strains has been attributed to a truncation mutation in RocA, a multifunctional regulator in GAS (2). We identified six single nucleotide polymorphisms (SNPs) in M3 *rocA* in comparison with *rocA* in M1 and, importantly, a single A deletion in a poly(A) septamer tract at nucleotide 1228. This single nucleotide deletion induced a premature stop codon after 1,251 bp, resulting in a truncated protein of 416 amino acids rather than 451 amino acids (Fig. 1). The truncation disrupts the hypothetical ATPase domain, which suggested that it may have an impact on RocA activity. We have since confirmed that the single A deletion is conserved in 448 United Kingdom M3 isolates, representing strains from the 1930s and contemporary isolates from the 1980s to the present day (A. Al-Shahib, A. Underwood, B. Afshar, C. E. Turner, T. Lamagni, M. Holden, S. Sriskandan, and A. Efstratiou, unpublished). The mutation is also present in the two existing NCBI sequences of M3 GAS originating from the United States (MGAS315) and Japan (SSI-1).

**FIG 2:**
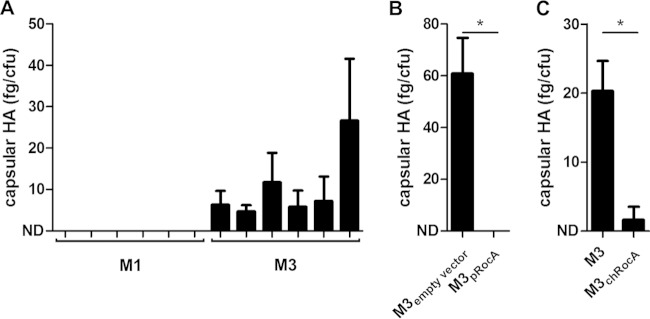
Serotype M3 capsule expression is derepressed by RocA M3 truncation. (A) M3 GAS express more capsule than M1 (*n* = 5 strains/group); however, capsule levels in M3 GAS were reduced by overexpression of full-length RocA (B) or chromosomal integration of a single copy of full-length RocA (C). All strains were wild type for CovR/S. M3 strains contained serotype-specific mutations in *fasC*, *rivR*, and *rocA*; M1 strains were wild type for these regulators. Data represent means (plus standard deviations) of results from 3 or 4 individual experiments. ND, not detected (below the limit of detection); *, Mann-Whitney *P* < 0.05.

We first corroborated the observation of Cao et al. and demonstrated that our M3 GAS expressed more capsule than M1 GAS, as expected (Fig. 2A). Subsequently, we showed that overexpression of full-length RocA amplified from M89 GAS (RocA_M89_) (2) was sufficient to reduce capsule expression to levels similar to those in M1 GAS (Fig. 2B). Furthermore, expression of a single copy of full-length *rocA*_M89_ incorporated into the M3 chromosome reduced levels of capsule expression, demonstrating that the unique mutations in serotype M3 RocA underlie the heightened levels of capsule expression exhibited by strains of this serotype (Fig. 2C). These data suggest that, in conjunction with the mutations in *fasC* and *rivR* identified by Cao et al., a truncation mutation in RocA may contribute to the association of M3 GAS with severe invasive infections and high mortality.
